# Sensor for In-Motion Continuous 3D Shape Measurement Based on Dual Line-Scan Cameras

**DOI:** 10.3390/s16111949

**Published:** 2016-11-18

**Authors:** Bo Sun, Jigui Zhu, Linghui Yang, Shourui Yang, Yin Guo

**Affiliations:** State Key Laboratory of Precision Measuring Technology and Instruments, Tianjin University, Tianjin 300072, China; bosun@tju.edu.cn (B.S.); jiguizhu@tju.edu.cn (J.Z.); shouruiyang@tju.edu.cn (S.Y.); yin_guo@tju.edu.cn (Y.G.)

**Keywords:** 3D shape measurement, line-scan cameras, structured light, large-scale metrology, industrial inspection

## Abstract

The acquisition of three-dimensional surface data plays an increasingly important role in the industrial sector. Numerous 3D shape measurement techniques have been developed. However, there are still limitations and challenges in fast measurement of large-scale objects or high-speed moving objects. The innovative line scan technology opens up new potentialities owing to the ultra-high resolution and line rate. To this end, a sensor for in-motion continuous 3D shape measurement based on dual line-scan cameras is presented. In this paper, the principle and structure of the sensor are investigated. The image matching strategy is addressed and the matching error is analyzed. The sensor has been verified by experiments and high-quality results are obtained.

## 1. Introduction

Three-dimensional (3D) shape measurement is widely utilized in various industries, including automobile, shipbuilding, aviation, and aerospace. The integration of 3D measurement in industrial development and production processes has benefits on improving overall quality assurance, speeding up the time to production, requiring less time and fewer personnel, and considerably increasing performance, all of which facilitate the optimization of the entire manufacturing process. Tremendous efforts have been devoted to 3D shape measurement and numerous methods have been developed [1,2]. Structured light techniques are most applied in industrial inspection due to their high accuracy, non-invasiveness, non-contact, and flexibility. However, existing methods meet great challenges as a result of the increasing demands for accuracy, volume, and speed. In certain applications, very large objects, such as turbine blades, metal hulls, airplane wings, and so forth, must be measured with rather high accuracy. Whilst the measurement tasks permit extremely short time in manufacturing conditions, or even require real-time measurement in the shop floor unit. Another challenge is that the measuring object keeps moving continuously, at a high speed in the production pipeline, such as quality inspection on a conveyor belt, inline measurement of a car body on the manufacturing line, and so on. Measurement must be done during the moving process. Up to now, few of existing techniques can resolve the above problems perfectly.

The point-wise structured light technique has the most significant advantages in its accuracy, and its relative insensitivity to illumination conditions and surface texture effects. The key limitation is its low-efficiency scanning mode where the laser beam must scan over the surface point-by-point. Similarly, the scanning speed of structured laser stripe technique limits to the camera’s frame rate and the image processing speed. To overcome such limitations, the fringe projection technique is proposed [3]. Multi-camera systems combining phase-shift with photogrammetry open the possibility for precise and fast 3D shape measurement. The greater redundancy and calibration-free projector promise a higher accuracy. Recent trends in the development of cameras and projectors allow high-speed acquisition of surfaces [4,5,6]. For example, the digital image correlation (DIC) systems are used for deformation, vibration, and strain measurement in high-speed transient and dynamic applications where the measured surfaces are constantly changing in real-time [7]. Although compelling results have been obtained, matrix cameras still have shortcomings in continuous measurement of large-scale objects or high-speed moving objects. First, continuous image acquisition in the moving process using matrix cameras demands ultimate synchronization. The result can hardly be truly continuous for high-speed moving objects. Second, matrix cameras have to obtain a complete surface with multiple standpoints. It is difficult to measure a large-scale object within a short time. Third, matrix cameras have to make a trade-off between resolution and frame rate. Finally, sophisticated high-speed matrix cameras and projectors are quite expensive and relatively delicate.

Line-scan cameras, as one-dimensional (1D) imaging sensors, provide both high resolution and high line rate (up to 16,384 pixels at 72 KHz [8]). Additionally, their lateral resolution is not limited to the sensor format, but comes from the scanning rate. Images can be simultaneously captured by scanning over objects. In summary, line-scan cameras have advantages of superior high resolution, seamless acquisition, greater and easily extendable measurement volume, and freedom of movement during measurement. Another advantage is its low-cost and simple structure. All of these features make line-scan cameras ideal for continuous measurement with high speed and high accuracy.

More recently, studies on 3D shape measurement using line-scan cameras have been reported. A line-scan camera-based stereo method for a color reconstruction of cultural heritage is described in [9]. The method provides a high-resolution result, but neither the absolute accuracy nor the point cloud density fulfills the demands of industrial applications, owing to its two-dimensional feature-based stereo correspondence method. In [10], a one-dimensional correlation method for searching corresponding points is employed. However, the result predominantly depends on surface texture. A structured light system using a single line-scan camera for measuring complex-shaped sheet metal parts is presented in [11]. The result is affected by the phase error caused by both the phase extraction algorithm and the surface contamination. A structured light method using dual line-scan cameras is proposed in [12], which is quite suitable for industrial applications because of its rapid, continuous, robust, and accurate result. The method would contribute to high-speed continuous measurement in industrial applications. Yet the systems are empirically designed by means of applying and simplifying the methodology of matrix cameras to the one-dimensional case, which is only satisfied under ideal conditions. The methodology of 3D shape measurement based on line-scan cameras is still somewhat mysterious and remains to be further investigated.

The general objective of our research is to provide a sensor for in-motion continuous 3D shape measurement based on dual line-scan cameras, aiming for precise and fast measurement of large-scale or moving objects in industrial applications. This paper focuses on the principle, structure, and error analysis of the sensor. The remaining of this paper is organized as follows: Section 2 describes basic theory of the sensor, including the triangulation principle of line-scan cameras and the stereo configuration; Section 3 presents the image matching strategy and analyzes the matching error; The experimental results are reported in Section 4; and final conclusions are summarized, and future research is presented, in Section 5.

## 2. 3D Shape Measurement Based on Line-Scan Cameras

Surface measurement deals with resolving the 3D coordinates of points that make up the object surface. In order to obtain a complete surface, a large number of object points (in the limit, each pixel) must be determined. The first key technology is triangulation of line-scan cameras, which determines the 3D coordinates of each point. Another key technology is the stereo configuration of the sensor, which arranges the cameras optimally to facilitate a high-speed measurement. Both key technologies are presented in the following subsections, respectively.

### 2.1. Triangulation of Line-Scan Cameras

**Figure 1:**
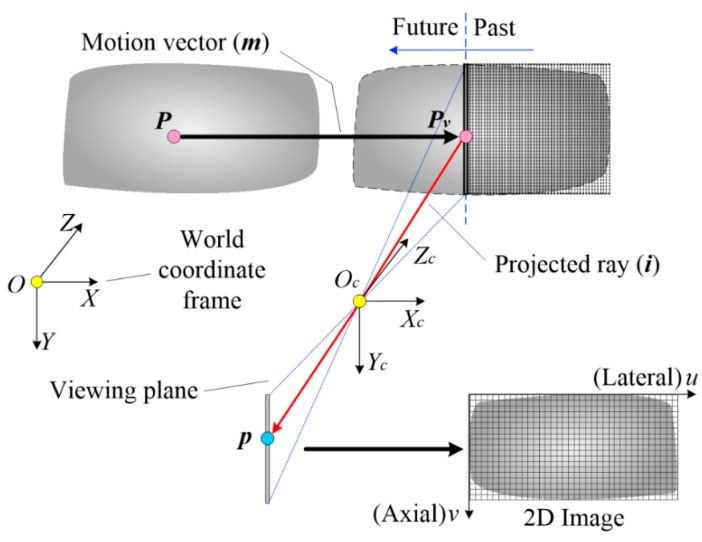
Imaging model of a line-scan camera.

A line-scan camera can be regarded as a special camera that consists of only one array of pixels at the center position. Perspective projection is only satisfied along the axial direction. As depicted in Figure 1, the perspective center and the sensor array determine a viewing plane, in which a ray from an object point ***P_v_*** to a corresponding image point ***p*** is described by collinearity equations:

(1) $$\left\{ \begin{array}{c} 0=r_{11}\left(X_{v}-X_{0}\right)+r_{12}\left(Y_{v}-Y_{0}\right)+r_{13}\left(Z_{v}-Z_{0}\right)\\ v=v_{c}+\Delta v+F_{y}\frac{r_{21}\left(X_{v\;}-\;X_{0}\right)\;+\;r_{22}\left(Y_{v}\;-\;Y_{0}\right)\;+\;r_{23}\left(Z_{v}\;-\;Z_{0}\right)}{r_{31}\left(X_{v}\;-\;X_{0}\right)\;+\;r_{32}\left(Y_{v}\;-\;Y_{0}\right)\;+\;r_{33}\left(Z_{v}\;-\;Z_{0}\right)} \end{array}.\right.$$

These equations describe the transformation of object coordinates (*X_v_*, *Y_v_*, *Z_v_*) into corresponding image coordinates (0, *v*) as a function of the intrinsic parameters (*v_c_*, *F_y_*) and extrinsic parameters (*X*_0_, *Y*_0_, *Z*_0_, *ω*, *φ*, *κ*). The subscripts *v* mean that the point belongs to the viewing plane. The first part is the viewing plane and the second part represents the perspective projection. The distortion *∆v* can be noted as follows:

(2) $$\Delta v=k_{1}{\left(v-v_{c}\right)}^{5}+k_{2}{\left(v-v_{c}\right)}^{3}+k_{3}{\left(v-v_{c}\right)}^{2},$$

where *k*_1_, *k*_2_, and *k*_3_ are distortion coefficients.

The imaging model described in Equation (1) is only applicable to the points belonging to the viewing plane. In order to obtain a complete scanning image by successive acquisition of multiple lines, a movement between the camera and the object is essential. The movement can be either from the camera or the object. In general, the movement comes from the camera while scanning very large objects. Alternatively, the movement comes from the objects in the measurement of fast-moving objects. Since the movement of the objects is equivalent to the case where the camera moves towards the opposite direction, all of the methods and conclusions are applicable to both cases. Here we make an assumption that the movement described in this paper is specified in terms of the object’s motion. The camera will image the object slice by slice, generating a sequence of (1D) image lines. These image lines stitch together, resulting in a 2D image, in which the coordinate *u* is proportional to the time a point takes to appear in the viewing plane:

(3) u=Ft,

where *F* is the line rate. As sketched in Figure 1, a point ***P***(*X*, *Y*, *Z*) appears in the viewing plane after traveling by time *t*, resulting in a projected point ***p***(*u*, *v*). The point moves by a vector ***m***(*t*) (*m_x_*(*t*), *m_y_*(*t*), *m_z_*(*t*)) and has new coordinates:

(4) $${\left[\begin{array}{ccc} X_{v} & Y_{v} & Z_{v} \end{array}\right]}^{T}={\left[\begin{array}{ccc} X & Y & Z \end{array}\right]}^{T}+{\left[\begin{array}{ccc} m_{x}\left(t\right) & m_{y}\left(t\right) & m_{z}\left(t\right) \end{array}\right]}^{T},$$

By substituting Equations (3) and (4) into Equation (1), we obtain the in-motion imaging model for all points:

(5) $$\left\{ \begin{array}{c} 0=r_{11}\left(X+m_{x}\left(t\right)-X_{0}\right)+r_{12}\left(Y+m_{y}\left(t\right)-Y_{0}\right)+r_{13}\left(Z+m_{z}\left(t\right)-Z_{0}\right)\\ v=v_{c}+\Delta v+F_{y}\frac{r_{21}\left(X\;+\;m_{x}\left(t\right)\;-\;X_{0}\right)\;+\;r_{22}\left(Y\;+\;m_{y}\left(t\right)\;-\;Y_{0}\right)\;+\;r_{23}\left(Z\;+\;m_{z}\left(t\right)\;-\;Z_{0}\right)}{r_{31}\left(X\;+\;m_{x}\left(t\right)\;-\;X_{0}\right)\;+\;r_{32}\left(Y\;+\;m_{y}\left(t\right)\;-\;Y_{0}\right)\;+\;r_{33}\left(Z\;+\;m_{z}\left(t\right)\;-\;Z_{0}\right)} \end{array},\right.$$

**Figure 2:**
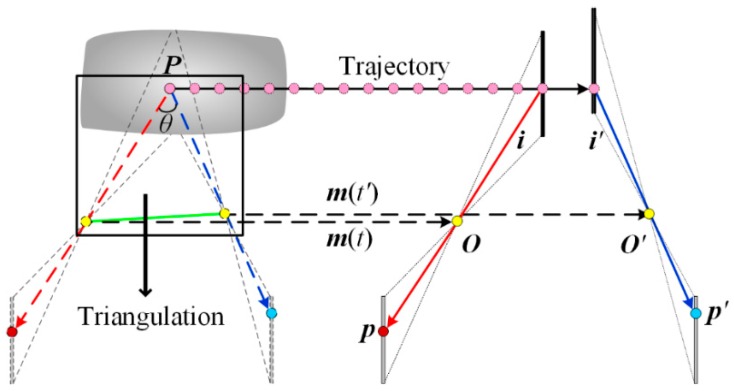
Triangulation of dual line-scan cameras.

In the viewing plane, the direction to the point ***P_v_*** is determined by the projected ray ***i*** from the perspective center ***O*** to the image point ***p***. However, its absolute position is still unknown. In order to compute the 3D coordinates of the point ***P***, this spatial direction must intersect with a second ray. Figure 2 shows the triangulation model of dual line-scan cameras. The point ***P*** keeps moving along its trajectory and appears in the viewing plane of a second camera after time *t’*, resulting in a second projected point ***p’***(*u’*, *v’*). The triangulation is highlighted in the black rectangle. The coordinates of the point ***P*** can be determined by solving following equations:

(6) $$\left\{ \begin{array}{c} 0=r_{11}\left(X+m_{x}\left(t\right)-X_{0}\right)+r_{12}\left(Y+m_{y}\left(t\right)-Y_{0}\right)+r_{13}\left(Z+m_{z}\left(t\right)-Z_{0}\right)\\ v=v_{c}+\Delta v+F_{y}\frac{r_{21}\left(X\;+\;m_{x}\left(t\right)\;-\;X_{0}\right)\;+\;r_{22}\left(Y\;+\;m_{y}\left(t\right)\;-\;Y_{0}\right)\;+\;r_{23}\left(Z\;+\;m_{z}\left(t\right)\;-\;Z_{0}\right)}{r_{31}\left(X\;+\;m_{x}\left(t\right)-\;X_{0}\right)\;+\;r_{32}\left(Y\;+\;m_{y}\left(t\right)\;-\;Y_{0}\right)\;+\;r_{33}\left(Z\;+\;m_{z}\left(t\right)\;-\;Z_{0}\right)}\\ 0={r_{11}}^{\prime }\left(X+m_{x}\left(t^{\prime }\right)-X_{0}^{\prime }\right)+{r_{12}}^{\prime }\left(Y+m_{y}\left(t^{\prime }\right)-Y_{0}^{\prime }\right)+{r_{13}}^{\prime }\left(Z+m_{z}\left(t^{\prime }\right)-Z_{0}\prime \right)\\ v^{\prime }={v_{c}}^{\prime }+\Delta v^{\prime }+{F_{y}}^{\prime }\frac{{r_{21}}^{\prime }\left(X\;+\;m_{x}\left(t^{\prime }\right)\;-\;X_{0}^{\prime }\right)\;+\;{r_{22}}^{\prime }\left(Y\;+\;m_{y}\left(t^{\prime }\right)\;-\;Y_{0}^{\prime }\right)\;+\;{r_{23}}^{\prime }\left(Z\;+\;m_{z}\left(t\prime \right)\;-\;Z_{0}\prime \right)}{{r_{31}}^{\prime }\left(X\;+\;m_{x}\left(t^{\prime }\right)\;-\;X_{0}^{\prime }\right)\;+\;{r_{32}}^{\prime }\left(Y\;+\;m_{y}\left(t^{\prime }\right)\;-\;Y_{0}^{\prime }\right)\;+\;{r_{33}}^{\prime }\left(Z\;+\;m_{z}\left(t^{\prime }\right)\;-\;Z_{0}\prime \right)} \end{array}\right.$$

where the coefficients with superscript apostrophe are the parameters of the second camera. Techniques for the calibration of the parameters are presented in [13,14,15].

### 2.2. Stereo Configuration of the Sensor

In theory, a point can be located as long as the intersection angle (*θ* in Figure 2) is nonzero. However, in order to realize precise and fast measurement, the stereo configuration of the sensor must satisfy certain criteria. A first criterion is to establish the stereo correspondences with less computation. It is vitally important because establishing millions of correspondences for the complete object surface is quite time-consuming. The problem is particularly severe for line-scan cameras due to their extremely high resolution and line rate. The required processing power increases rapidly as the resolution and frame rate increase. A second criterion is that the stereo configuration should be compatible with a suitable structured light illumination solution, which is necessary to obtain a precise and robust result. Hence, both criteria should be considered to optimize the stereo configuration, aiming for high-accuracy and high-speed measurement.

Correspondence problem is an essential subject in 3D shape measurement. The existence of epipolar geometry can significantly reduce the search space for matching homogeneous points. However, the epipolar geometry of line-scan cameras is quite different from that of matrix cameras. The main problem stems from the imaging mechanism of line-scan cameras. The major characteristic of line-scan imaging is that each individual line has its own exposure time. Such a feature will affect the epipolar geometry of line-scan cameras.

**Figure 3:**
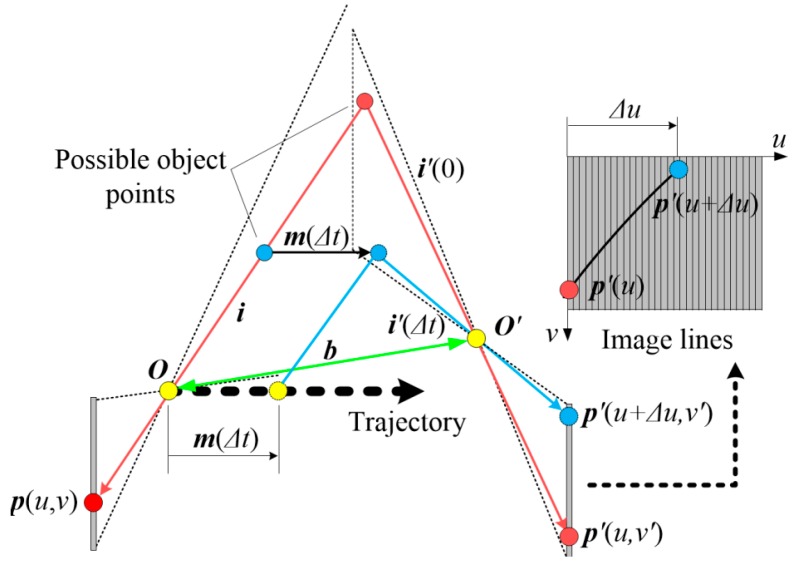
Locus of all possible conjugate points to a selected point.

The epipolar line in the right image can be regarded as the locus of all possible conjugate points to a selected left image point ***p***(*u*, *v*). As shown in Figure 3, such a locus can be derived by changing the depth of the corresponding object point along the projected ray ***i*** that connects the perspective center ***O*** and the image point ***p***. The projected ray moves simultaneously with the object point along the trajectory, intersecting with the right viewing plane after time interval ∆*t.* The intersection point and the perspective center ***O’*** determine another projected ray that is denoted as ***i’***(*∆t*). The ray projects onto the right camera at an image point ***p’***(*u’*, *v’*). The line difference between two image points is noted as:

(7) $$\Delta u=F\Delta t=u^{\prime }-u,$$

For different object points, the projected rays vary accordingly and result in different image coordinates. According to the conclusions from [16,17], by assuming a linear movement and constant orientation, the locus of the image point ***p’***(*u’, v’*) satisfies the relationship:

(8) $$\left[\begin{array}{cccc} u^{\prime } & u^{\prime }v^{\prime } & v^{\prime } & 1 \end{array}\right]\left[\begin{array}{cccc} 0 & 0 & f_{13} & f_{14}\\ 0 & 0 & f_{23} & f_{24}\\ f_{31} & f_{32} & f_{33} & f_{34}\\ f_{41} & f_{42} & f_{43} & f_{44} \end{array}\right]\left[\begin{array}{c} u\\ uv\\ v\\ 1 \end{array}\right]=0,$$

where the coefficients *f_ij_* (*i*, *j* = 1, 2, 3, 4) can be expressed as functions of the cameras’ parameters and the movement parameters. The epipolar geometry of line-scan cameras is derived from Equations (7) and (8):

(9) $$A_{1}\Delta u+A_{2}v^{\prime }+A_{3}\Delta uv^{\prime }+A_{4}=0,$$

where *A*_1_, *A*_2_, *A*_3_, and *A*_4_ can be denoted as functions of the coefficients *f_ij_* (*i*, *j* = 1, 2, 3, 4). Owing to the existence of the third term *A*_3_, the epipolar constraint is not a line, but a hyperbola, in most cases.

The benefit in using such constraint is to cut down the search space. However, it is not enough for high-speed measurement because calculating the hyperbola equations for all pixels requires a significant amount processing power and time. Fortunately, it is easy to customize experimental arrangements in industrial applications. The following two ideal stereo configurations can simplify the correspondence problem, without having to calculating the epipolar constraint.

● Configuration 1

**Figure 4:**
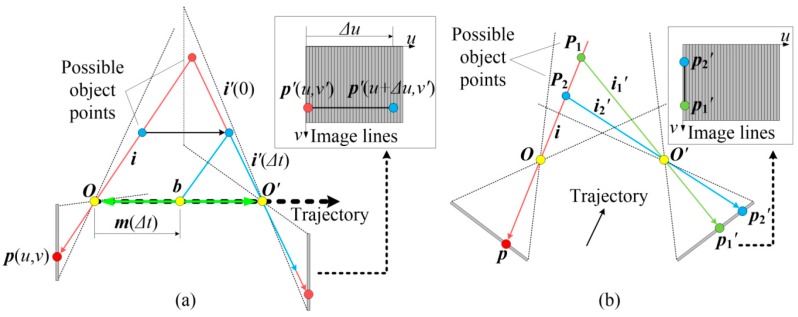
Ideal stereo configurations. (**a**) Direction of the trajectory is always consistent with the base vector; and (**b**) the two viewing planes are coplanar.

As demonstrated in Figure 4a, the direction of trajectory is always consistent with the base vector (that is ***m***(*∆t*)//***b***). The projected rays ***i’***(*∆t*) are always the same and project onto the right camera at a same pixel. As a consequence, the epipolar line is along lateral direction (*u* axis).

● Configuration 2

Alternatively, the cameras must be arranged in such a way that the viewing planes are coplanar, as shown in Figure 4b. The correspondences exist within the same lines. As a result, the epipolar line is along the axial direction (*v* axis).

The former has the advantage of providing a large overlapping field of view (FOV). Almost the entire FOVs are overlapped, which would contribute to a larger measurement volume. In addition, it is convenient to arrange the sensor under the actual measurement field, since the space along the movement direction is generally broad. However, such a configuration requires precise movement that is hard to achieve in several applications because it is relatively difficult to provide an ideal motion under some harsh conditions of the industrial environment where there are many restrictions on experimental arrangements. By contrast, the latter configuration has no requirements on movement. On the other hand, it must be noted that industrial measurement usually utilizes structured light techniques to enhance the surface texture. For the former configuration, the cameras cannot guarantee to capture all points concurrently. Developing a projection solution for such configuration is a key challenge. If this point is dealt with well, the former configuration is quite promising and has significant advantages. Anyway, establishing correspondences for such a configuration is interesting and challenging. Conversely, it is easy to utilize structured light for the latter configuration because the cameras always capture objects synchronously. To be honest, we have not found a good illumination solution for the former configuration up to now. Accordingly, the proposed sensor in this paper adopts the latter configuration.

## 3. Image Matching Strategy and Matching Error Analysis

### 3.1. Structured Light Solution

As mentioned previously, the objects under measurement in industrial applications usually does not provide sufficient texture, thus the structured light technology plays a critical role to ensure accurate and robust results. Various structured light techniques are introduced in [18,19,20]. As line-scan cameras are in-motion during image acquisition, sequential projection techniques, such as binary code, gray code, and phase shift, cannot be established in theory. In contrast, single-shot projection techniques can avoid such errors due to a single pattern being enough to perform the image matching. Therefore, single shot projections are more suitable for the sensor. More recently, the programmable projectors allow projection of binary patterns at a rate of up to 4 to 10 kHz [21]. In this regard, the pattern rate has already been fast enough. However, the brightness under such a high rate is still too low to produce high-quality results. Therefore, a sensible strategy for the sensor is to adopt static pseudo-random binary pattern. Another reason for not employing continuous varying patterns will be described in Section 3.3.

### 3.2. Real-Time Correlation Method

In accordance with the description in Section 2.2, if the viewing planes are aligned coplanar, homogeneous image points will be distributed in the same lines. Besides, the images are captured line by line. There is no correlation in the lateral direction. A simplified one-dimensional normalized cross correlation (NCC) method is utilized by the sensor. The cross-correlation of a left sub-window ***L*** centered at (*u*, *v*) with a right sub-window ***R*** centered at (*u*, *v’*) is:

(10) $$C\left(u,v,v^{\prime }\right)=\frac{\sum_{i=-n}^{n}\left[L\left(u,\;v\;+\;i\right)-\bar{L}\right]\left[R\left(u,v^{\prime }\;+\;i\right)-\bar{R}\right]}{\sqrt{\sum_{i=-n}^{n}{\left[L\left(u,\;v\;+\;i\right)-\bar{L}\right]}^{2}\sum_{i=-n}^{n}{\left[R\left(u,v^{\prime }\;+\;i\right)-\bar{R}\right]}^{2}}},$$

where *n* is the half size of the sub-window, which should be determined such that the coded pattern of any sub-window is unique. The range of the search space is a function of the maximum depth in the object space. For each left image point *v*, the right image point *v’* with the maximum correlation is most likely to be the true position. The curve around the maximum is approximated by a quadratic function, so the desired position can be determined to sub-pixel precision.

In order to implement high-speed measurement, a graphics processing unit (GPU) is used by the sensor for the massive computations for both image matching and triangulation. A GPU contains a large number of streaming processors that operate concurrently and, therefore, allows effective parallelism of tasks with little computational dependency. Each processor independently computes the correlation and depth for each single pixel. The lack of computational dependency makes this operation extremely fast, which, in general, can yield several orders of magnitude higher performance than a conventional central processing unit (CPU). With a powerful GPU and an optimized program, the image matching and the triangulation algorithms are able to run in real-time.

### 3.3. Matching Error against Non-Coplanar Viewing Planes

**Figure 5:**
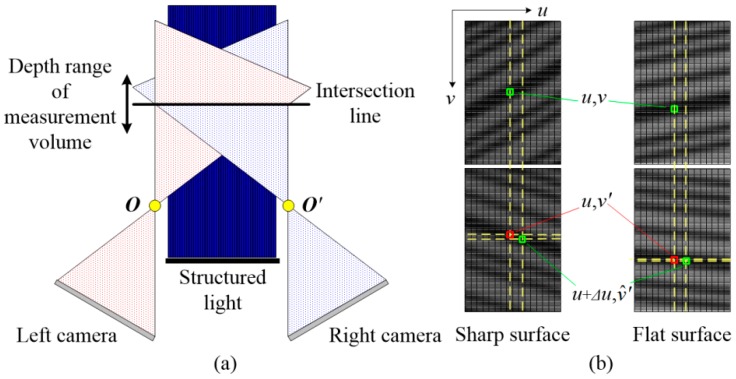
Matching error against non-coplanar viewing planes. (**a**) Misalignment of two viewing planes; and (**b**) schematic drawing of the matching error.

The correspondence between homogeneous image points resides in the same lines under ideal condition. However, aligning the cameras coplanar exactly is easier said than done, and usually costly in time and personnel. The alignment, especially the orientation alignment, needs not only precise devices but also sophisticated skills. Furthermore, such precision would not last long owing to the strong impulse or severe acceleration during long-term running in the harsh environmental condition of industrial shop floor. Therefore, non-coplanarity exists, inevitably, in spite of precise adjustment. Figure 5a shows the non-coplanar condition. The viewing planes intersect at a straight line and only points on this line can be captured concurrently. The points outside this line would project on different lines. This is another reason for not employing continuous varying patterns that are not able to work in non-ideal cases. By ignoring the difference, the sensor still searches correspondences along the axial direction and then calculates triangulation according to Equation (6). As shown in Figure 5b, for a left image point ***p***(*u*, *v*), the output of the correlation algorithm is ***p’***(*u*, *v’*), yet the factual position is ${\hat{p}}^{\prime }$(*u* + ∆*u*, ${\hat{v}}^{\prime }$). The line difference ∆*u* is the lateral error and the difference between *v’* and ${\hat{v}}^{\prime }$ is the axial error. The axial error is essentially caused by the lateral error owing to the operating mechanism of the matching strategy. The axial error will be larger for a sharp surface and smaller for a flat surface because the gradient (difference between adjacent lines) of the pattern increases with the surface curvature. Both lateral and axial errors would lead to errors in the final surface.

Diminishing the misalignment is crucial for the sensor. As mentioned above, aligning the cameras in an exactly coplanar manner is difficult and unnecessary. In practice, the cameras just need to be aligned to an acceptance level where the matching accuracy can be tolerated and maintained. The acceptance level can be determined by simulating the lateral error against the misalignment.

The factual positions of homogeneous image points must follow the epipolar constraint in Equation (9). However, the calculated positions reside in the same lines, which means ∆*u* should always be zero. Thus, Equation (9) is also the error curve. The curve for a given left pixel can be denoted as a function:

(11) $$\Delta u=f\left(v^{\prime }\right).$$

For a given stereo configuration, points with equal *v* coordinates would share an identical error curve. There are *N* (resolution of the left camera) curves in total for all pixels:

(12) $$\Delta u_{v}=f_{v}\left(v^{\prime }\right),\;\mathrm{for}\;v=0,1,\ldots ,\;N-1.$$

Equations (11) and (12) demonstrate that the lateral error for a point depends on both its *v* coordinate and its depth. Points with equal *v* coordinates do not necessarily have equal lateral errors due to their different depths. Since different depths will lead to different disparities that cause different *v’* coordinates, finally resulting in different lateral errors. Only if the *v* coordinates and depths are both the same, the lateral errors will be equal.

**Figure 6:**
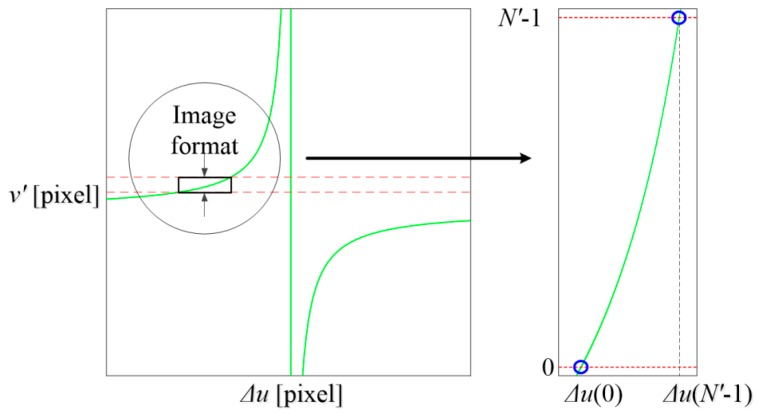
Epipolar curve for a given left pixel value.

Figure 6 illustrates one curve. Since the curve is continuous and is truncated by the image format of the right camera, the maximum error exists at either the first or the last pixel:

(13) $$E\left(v\right)=max\left[\left|f_{v}\left(0\right)\right|,\left|f_{v}\left(N^{\prime }-1\right)\right|\right],\;\mathrm{for}\;v=0,\;1,\ldots ,N-1.$$

where *N’* is resolution of the right camera. A total of *N* errors are obtained and the maximum absolute value is defined as the maximum lateral error:

(14) $$E_{M}=\underset{0\leq v\leq N-1}{max}\left[\left|E\left(v\right)\right|\right].$$

**Figure 7:**
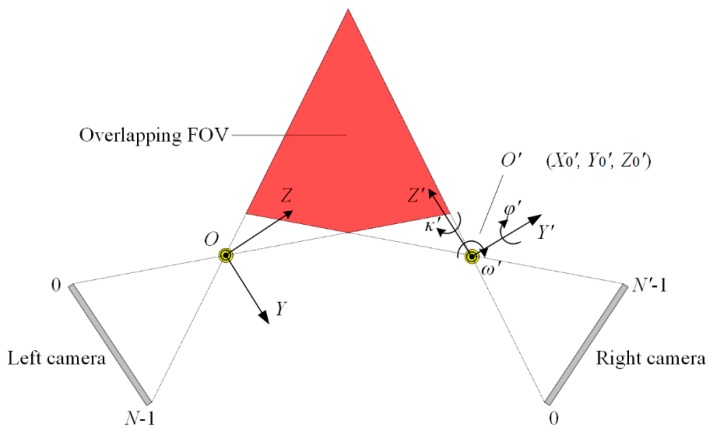
Simulation of the lateral error for the sensor.

Taking the actual experimental system as an example, the parameters are listed in Table 1. The left camera is assumed to be aligned to the world coordinate frame, as shown in Figure 7, so the parameters *ω’*, *Y*_0_*’*, and *Z*_0_*’* would not contribute to the misalignment, and the parameters *φ’*, *κ’*, and *X*_0_*’* (should be zero ideal) are the error sources.

**Figure 8:**
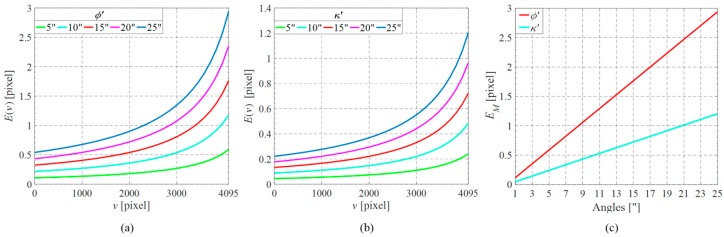
Simulation results of lateral errors with respect to various parameters. (**a**) Lateral errors with respect to the misalignment angle *φ’*; (**b**) lateral errors with respect to the misalignment angle *κ’*; and (**c**) the maximum lateral errors with respect to the misalignment angles *φ’* and *κ’*.

The maximum lateral errors with respect to various misalignment angles of *φ’* and *κ’* are estimated according to the above analysis. Both angles vary from 1–25 arcsec. Figure 8 shows the results where the lateral error increases with both misalignment angles. It can be concluded by comparing Figure 8a,b that the lateral error is more sensitive to *φ’* than *κ’*. Figure 8c shows that the angles *φ’* and *κ’* should be smaller than 9 and 21 arcsec, respectively, to ensure the maximum lateral error remain less than one pixel. In practice, the marginal pixels may not be used for triangulation because of the finite depth of the measuring object. Thus, the factual maximum lateral error is usually smaller than the computed value. The misalignment angles can be a little more tolerated. On the other hand, the lateral error with respect to *X*_0_’ is proportional to *X*_0_’ itself, independent of the right pixel *v’*:

(15) $$\Delta u=\left|\frac{F}{v_{x}}{X_{0}}^{\prime }\right|$$

Similarly, *X*_0_’ should be smaller than 0.12 mm, that is the ratio of *v_x_* and *F*.

Such error analysis has functions of estimating error distribution of real measurement, indicating the acceptance level for the coplanarity alignment, facilitating to better align the cameras by telling which parameter is more sensitive, and teaching us to choose the appropriate grade of adjustment equipment, which is cheaper, smaller in size, but precise enough for the coplanarity alignment.

## 4. Experimental Results

**Figure 9:**
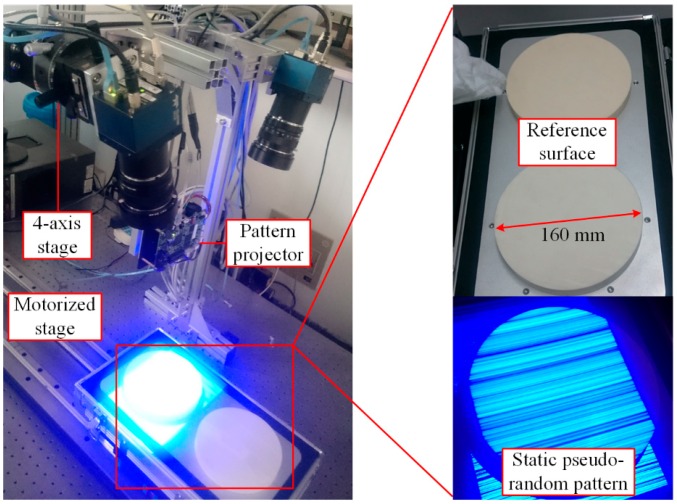
Experimental setup for verifying the sensor.

The sensor has been verified using the experimental setup shown in Figure 9. Two Spyder3 (Teledyne DALSA Inc., Waterloo, ON, Canada) line-scan cameras of 4096 pixels, with a pixel size of 10 μm and a focal length of 50 mm, are used. The cameras provide configurable I/O triggers for synchronous imaging. The frame rate of the sensor is set to 500 Hz. One camera is fixed and the other one is mounted on a four-axis stage that provides uncoupled tilt adjustment in pitch and roll, together with rotation (yaw) and height adjustment for the coplanarity alignment. According to the simulation results, the stage with a minimum incremental angle of 5 arcsec and a minimum incremental placement of 10 µm is precise enough for the alignment. Three reference surfaces are used to check the measurement quality of the sensor. The surfaces, including a flat surface, a concave spherical surface, and a convex spherical surface, made of zirconia (with flatness of 1 µm), are fixed onto a motorized stage by which a linear motion with constant velocity and orientation along a straight line is provided. A DLP LightCrafter 4500 (Texas Instruments Inc., Dallas, TX, USA) evaluation module is employed to generate the static pseudo-random pattern. Blue light is chosen for illumination because of its narrowband that enables precise measurements, independent of environmental lighting conditions. A workstation containing an eight-core Intel i7 CPU and a GTX Titan Black GPU is employed. The stereo-matching and triangulation algorithms run on both platforms to make a performance comparison.

**Figure 10:**
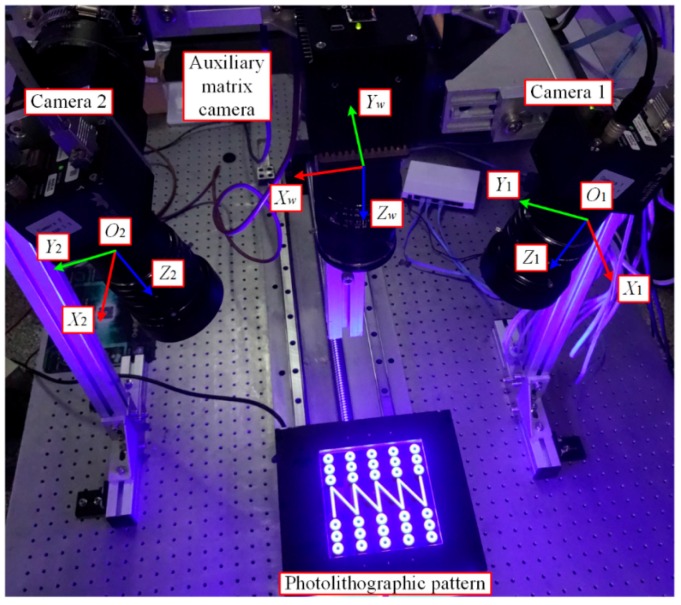
Calibration of the sensor.

The sensor is calibrated after the coplanar adjustment. As shown in Figure 10, an auxiliary matrix camera is used to aid the calibration. Detailed information about the calibration method is described in [15].

**Figure 11:**
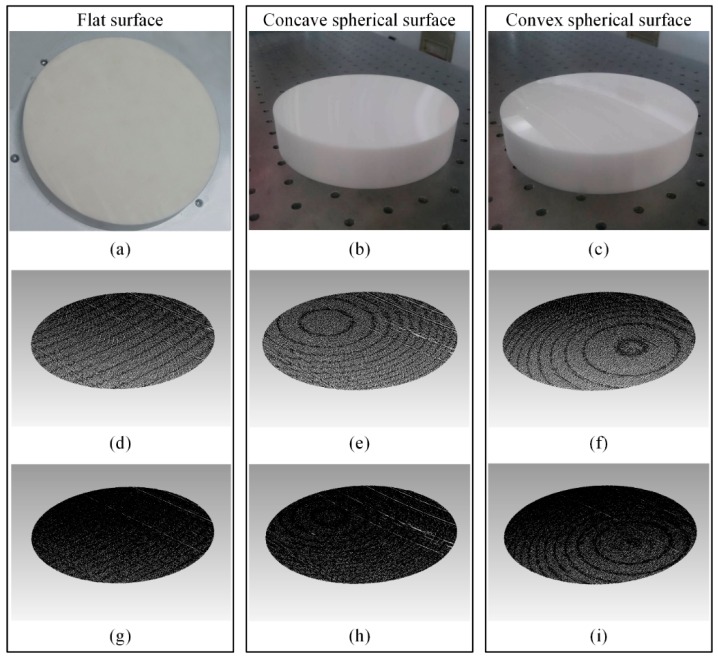
Reference surfaces and measured point clouds. (**a**–**c**) Reference surfaces; (**d**–**f**) measured point clouds with a speed of 60 mm/s; and (**g**–**i**) measured point clouds with a speed of 30 mm/s.

The sensor scans over the surfaces with speeds of 60 mm/s and 30 mm/s, respectively. A XL-80 (Renishaw Inc., Wotton-under-Edge, UK) interferometric straightness interferometer is employed to monitor the screw jitter of the motorized stage. The obtained point clouds are shown in Figure 11. The results with speeds of 60 mm/s and 30 mm/s are almost the same, except for the point cloud density.

Best-fit reference surfaces are fitted from the obtained point clouds. The deviations from each point to the reference surfaces are calculated. According to the description in [22], about 0.1% of the measured points from the raw data are eliminated because of the unavoidable outliers. The statistical results of the deviations are listed in Table 2. The first two columns are the experimental configurations. The third column is the screw jitter of the motorized stage, obtained from the interferometric straightness measurement. The last three columns are the maximum, minimum, and root-mean-square (RMS) values of the deviations. As can be seen, the overall quality of the results with 30 mm/s is a little better than the results with 60 mm/s. It is mainly because the motorized stage has slightly less screw jitter with a lower moving speed. The screw jitter will directly lead to position changes of all surface points. Thus, the overall quality will be affected. As listed in Table 2, the difference in the screw jitter is almost equal to the difference in RMS values.

**Figure 12:**
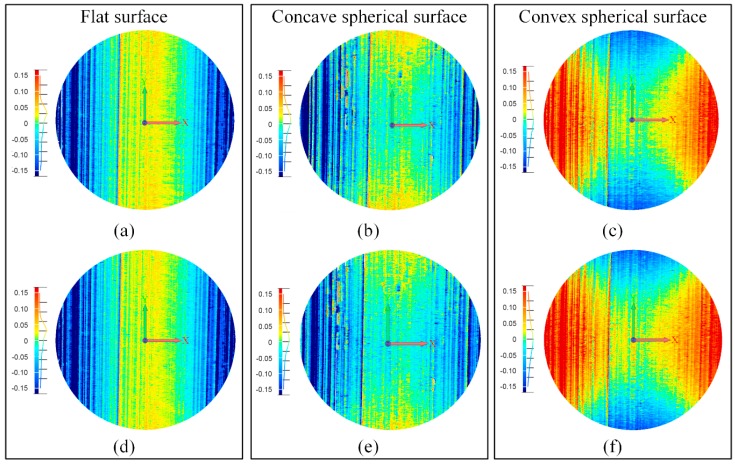
Color maps of the deviations from each measured point to the best-fit surfaces. (**a**–**c**) Color maps of surfaces with a speed of 60 mm/s; and (**d**–**f**) color maps of surfaces with a speed of 30 mm/s.

The color maps of the deviations are shown in Figure 12. The color maps with different speeds are almost the same. Stripes along the *y* direction can be clearly seen in all color maps. In fact, the surface error comes from the calibration error, the screw jitter, and the matching error. Among them, the calibration error is less than 0.1 pixel, which can be ignored. The error caused by the screw jitter is uniformly distributed along the surface. Thus, the color map of the surface error predominantly depends on the matching error. For the smooth measured reference artifacts, the matching error is mainly determined by the lateral error. According to the error analysis in Section 3.3, points with equal *v* coordinates and depths have equal lateral errors. Therefore, the error distributions of the flat surface are uniform stripes owing to the almost identical depths. The error distributions of the concave and convex spherical surfaces are *x*-axial symmetrical stripes because of the symmetrical depths.

The matching results using the CPU and the GPU are reported in Table 3. The first two columns are the experimental configurations. The third and fourth columns are the matched pixel numbers, which are almost the same for both platforms. The last two columns are the processing speeds. The processing speeds of using the GPU are hundreds of times faster than using the CPU. As the pixel numbers increase, the processing speeds of using the CPU decrease and processing speeds of using the GPU remain unchanged. Actually, the processing speeds of the GPU can be further improved by optimizing the parallel programming approach, enabling maximum concurrency of the individual streaming processors.

## 5. Conclusions

A sensor for in-motion continuous 3D shape measurement based on dual line-scan cameras is presented in this paper. The basic principle and the stereo configuration are elaborated in detail. The image matching strategy is introduced and the matching error is analyzed. The sensor is verified by measuring reference artifacts. The results are compared with the nominal values of the reference artifacts, demonstrating a rather high accuracy. Depth data are calculated on both CPU and GPU platforms to compare the performance.

Comparing with conventional sensors based on matrix cameras, our proposed sensor is a promising technique for fast measurement of large-scale objects and high-speed moving objects. Compared with fringe projection sensors, our sensor has advantages of seamless acquisition, greater and easily extendable measurement volume, and freedom of movement during measurement. Another advantage is its low-cost and simple structure. Compared with structured laser sensors, a distinct advantage of our sensor is the superior high sample rate, which is very useful for shop floor inline measurement.

Although the sensor is only verified using a uniform linear motion, in fact, the sensor can also work with freedom of motion. All scans can be aligned to a complete surface by means of ancillary global-measuring devices, similar to the commercial systems, such as Leica T-Scan [23], Nikon K-Scan [24], Creaform MetraSCAN [25], etc. This paper just focuses on the principle, structure, and error analysis of the sensor. Integrating all scans without global-measuring devices will be the core of future research. Then more complicated and flexible motion and more measuring objects will be attempted. More motion platforms, including industrial robots and mobile robots, will be applied.

## Figures and Tables

**Table 1:** Parameters used for simulation of the lateral error.

***v_c_*/*N* (pixel)**	***F_y_* (pixel)**	***v_c_’*/*N’* (pixel)**	***F_y_’* (pixel)**	**ω*’* (°)**	***Y*_0_*’* (mm)**	***Z*_0_*’* (mm)**
2048/4096	5000	2048/4096	5000	50	400	75
***φ’* (”)**	***κ’* (”)**	***X*_0_*’* (μm)**	***v_x_* (mm/s)**	***v_y_* (mm/s)**	***v_z_* (mm/s)**	***F* (Hz)**
1 to 25	1 to 25	0	−60	0	0	500

**Table 2:** Statistical results of the deviations.

Measured Suface	Scanning Speed (mm/s)	Screw Jitter (μm)	Max (mm)	Min (mm)	RMS (mm)
Flat Surface	60	23	0.237	−0.581	0.072
	30	15	0.229	−0.535	0.062
Concave spherical surface	60	23	0.469	−0.348	0.073
	30	15	0.454	−0.591	0.059
Convex spherical surface	60	23	0.564	−0.761	0.076
	30	15	0.465	−0.822	0.068

**Table 3:** Matching results.

Measured Suface	Scanning Speed (mm/s)	CPU Pixels	GPU Pixels	CPU (Kpixel/s)	GPU (Mpixel/s)
Flat Surface	60	1,706,452	1,702,784	15.513	19.649
	30	3,421,450	3,425,468	11.367	19.328
Concave spherical surface	60	1,743,740	1,749,575	12.728	17.691
	30	3,478,198	3,479,648	9.275	17.259
Convex spherical surface	60	1,784,801	1,781,576	15.520	19.537
	30	3,562,053	3,569,705	7.727	18.761
